# The augmentation of cytotoxic immune cell functionality through physical exertion bolsters the potency of chemotherapy in models of mammary carcinoma

**DOI:** 10.1002/cam4.6951

**Published:** 2024-01-17

**Authors:** Bingqing Qin, Zhongshi He, Lixia Xie, Shenglan Feng, Junjie Ye, Jianjun Gui, Xiaodong Sun, Ming Sang

**Affiliations:** ^1^ Research Center for Translational Medicine, Department of Oncology, Hubei Provincial Clinical Research Center for Parkinson's Disease at Xiangyang No.1 People's Hospital, Hubei Key Laboratory of Wudang Local Chinese Medicine Research Hubei University of Medicine Shiyan China

**Keywords:** breast cancer, cell apoptosis, cytotoxic immune cells, doxorubicin, exercise training

## Abstract

**Background:**

Mammary carcinoma, a pervasive and potentially lethal affliction, is conjectured to be profoundly influenced by physical exercise, both in prophylaxis and therapeutic contexts. This study endeavors to explore the repercussions of exercise training on the progression of mammary carcinoma, particularly the mechanisms by which the amalgamation of an exercise regimen and doxorubicin induces tumor cell apoptosis.

**Methods:**

Female BALB/c mice were categorized into four distinct groups: A sedentary group (SED), an exercise group (Ex), a doxorubicin group (Dox, 5 mg/kg), and a combined treatment group (Dox + Ex). The exercise training lasted for 21 days and included aerobic rotarod exercise and resistance training. The impact of exercise training on tumor growth, immune cell proportions, inflammatory factor levels, and cell apoptosis pathway was assessed.

**Results:**

Exercise training significantly curtailed tumor growth in a mouse model of breast cancer. Both the Ex and Dox groups exhibited significant reductions in tumor volume and weight (*p* < 0.01) in comparison to the SED group, while the Dox + Ex group had a significantly lower tumor volume and weight than the Dox group (*p* < 0.01). Exercise training also significantly increased the proportion of NK and T cells in various parts of the body and tumor tissue, while decreasing tumor blood vessels density. Exercise training also increased IL‐6 and IL‐15 levels in the blood and altered the expression of apoptosis‐related proteins in tumor tissue, with the combined treatment group showing even more significant changes.

**Conclusions:**

Physical training improves the effectiveness of doxorubicin in treating breast cancer by activating cytotoxic immune cells, releasing tumor suppressor factors, and initiating mt‐apoptosis, all while mitigating the adverse effects of chemotherapy.

## 1 INTRODUCTION

Breast cancer exhibits the highest frequency of occurrence and stands as a prominent cause of mortality in the female population.^1^ Conventional therapeutic methods, such as surgery, radiation therapy, and chemotherapy, result in reduced immune function and an increased risk of recurrence and metastasis.^2^ Doxorubicin, a broad‐spectrum anti‐tumor drug, has shown efficacy against various malignancies.^3^ However, this drug is associated with multiple adverse reactions, with cardiac toxicity being a particularly severe concern.^4^ In addition to genetic factors, women's susceptibility to breast cancer can be influenced by occupational and emotional stress. Exercise can help patients manage their psychological well‐being, alleviate stress and emotional burdens, and enhance bone health.^5^ Furthermore, exercise has been shown to have benefits on cardiovascular function and immune response, inhibiting breast cancer progression and enhancing chemotherapy responsiveness,^6^, ^7^ which has sparked interest in uncovering the underlying molecular mechanisms. Importantly, physical exercise enhances the anti‐tumor activity of cytotoxic immune cells, such as natural killer (NK) cells and cytotoxic T lymphocytes (CTLs).^8^ Studies suggest that exercise reduces circulating concentrations of hormones and growth factors, including insulin‐like growth factor‐1 (IGF‐1), which are known to promote tumor growth.^9^ Additionally, exercise reduces inflammation, a critical driver of tumor progression and metastasis.^10^, ^11^ Moreover, physical activity enhances augmented blood circulation and oxygen supply to tissues, potentially facilitating the conveyance of anti‐tumor medications to tumor locations.^12^

The combination of exercise training with chemotherapy is expected to become a promising strategy for the management of breast cancer. The objective of this study was to clarify the molecular mechanisms dictating the anti‐tumor effects of physical activity, encompassing the activation of cytotoxic immune cells, remodeling of the tumor microenvironment, and initiation of apoptosis in tumor cells. Moreover, we are concerned about the potential of exercise in mitigating doxorubicin‐induced cardiotoxicity while enhancing its overall anticancer efficacy.

## 2 MATERIALS AND METHODS

### 2.1 Cell culture

4T1 mammary carcinoma cells (Catalog No. CL‐0007), sourced from Procell Life Science & Technology Co., Ltd. (Wuhan, China,), were cultured in RPMI 1640 medium supplemented with 10% fetal bovine serum (FBS, Capricorn, FBS‐HI‐11A), as well as 100 IU/mL penicillin G sodium and 100 mg/mL streptomycin sulfate. Cells were cultured in a 37°C incubator with 5% CO_2_ and 95% air humidity, utilizing those in the exponential growth phase for the experiments.

### 2.2 Animal experiments

This study received approval from the Ethical Committee for Animal Experimentation of Xiangyang No.1 People's Hospital (XYYYE20220030). Twenty‐four female BALB/c mice (14–20 g, four‐week‐old) (Beijing Vital River Laboratory Animal Technology Co., Ltd, Beijing, China) were housed at 20°C–22°C with 50%–60% relative humidity and fed with standard laboratory chow and tap water ad libitum. The animals were assigned random identification numbers. Then, to observe the tolerance of mice to exercise intensity, all mice received aerobic and resistance exercise training for 1 week after acclimatization feeding. On day 0, all mice received 4T‐1 cells (5 × 10^5^ for each one) injection in the 4th mammary fat pad to construct an orthotopic breast cancer model. Ear tags were applied to all mice following tumor transplantation. One week later, a computer‐generated random number sequence was used to assign the mice into four groups, sedentary (SED, *n* = 6), exercise (Ex, *n* = 6), doxycycline (Dox, *n* = 6), and doxycycline + exercise (Dox + Ex, *n* = 6). The Ex group, Dox group, and Dox + Ex group received treatment protocol. An overview of the experimental outline can be seen in Figure 2 A. Briefly, the mice in the Ex group and Dox + Ex group received continuous training from Monday to Friday and rest over the weekend, while the mice in the Dox group and Dox + Ex group were treated with doxycycline (5 mg/kg, 3 times/week) via intraperitoneal injection, all treatment last 3 weeks. Blinding procedures were implemented, with all the mice numbered and randomly grouped, except for the experimenter who performed the exercise training and doxycycline treatment on the mice, the group information corresponding to the mice code was not known to the participants in the subsequent experimental testing. Body weight and tumor size were evaluated every 3 days: V = (a × b^2^)/2 (a = larger tumor diameter (mm); b = small tumor diameter (mm)).^13^ After concluding the treatment, the mice wer euthanized, and subsequent detection involved the collection of peripheral blood, tumor tissues, and internal organs.

### 2.3 Mice exercise mode

**FIGURE 1:**
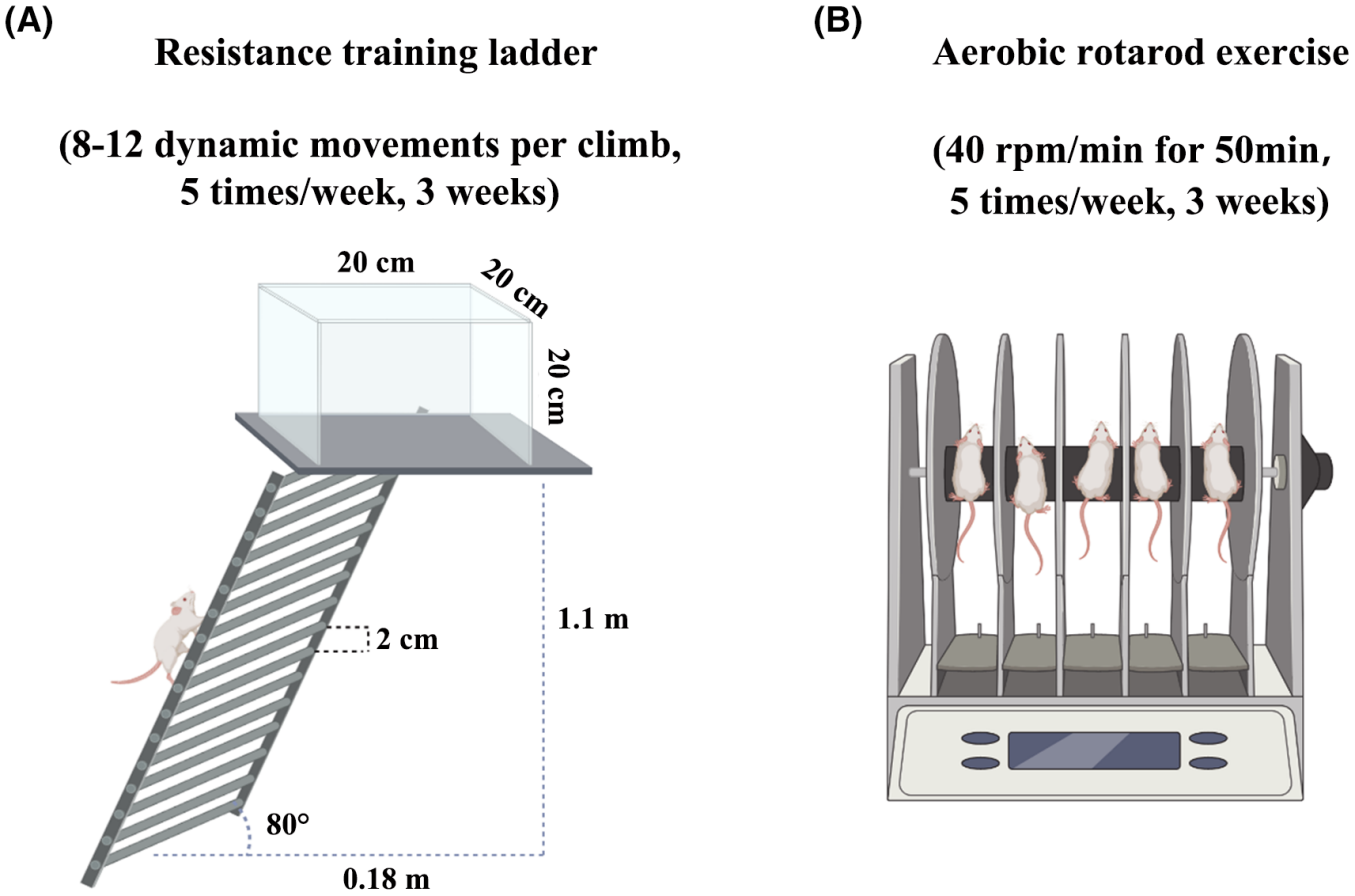
Exercise training design. (A) Resistance exercise. During the 21 days, climbing sessions were performed five times per week. The animals performed 8–12 dynamic movements per climb. (B) Aerobic exercise training. The running speed of medium‐intensity exercise is 40 rpm/min, and the exercise time is 50 min/day for 21 days.

Resistance training was carried out between 2:00 p.m. and 4:00 p.m. from Monday to Friday, with the experimental protocol following the schedule established by Hornberger and Farrar, and rest days designated for Saturday and Sunday.^14^ The mice were trained to ascend a ladder (1.1 m × 0.18 m, 2 cm intervals, inclined at 80°) and execute 8–12 dynamic movements per climb. The training device is shown in Figure 1A. The mice were trained for aerobic exercise using a rotating rod machine (Shanghai Xinruan Information Technology, China). Throughout the acclimatization phase, the rotational speed was progressively elevated from 4 rpm/min to 40 rpm/min until the mice could sustain continuous running at 40 rpm/min for a duration of 10 min without experiencing falls. Mice received a total of five aerobic exercise sessions per day from Monday to Friday (10 min/sessions, 2 min rest between sessions) and no training on weekends (Figure 1 B).^15^, ^16^ The mice were subjected to aerobic training 1 h before resistance training.

### 2.4 Histomorphometry

The heart, spleen, and tumor samples were fixed in 4% polyformaldehyde, paraffin‐embedded, and sectioned into 5 μm thick slices. Standard protocols were employed for Hematoxylin and Eosin (H&E) staining. Immunohistochemical (IHC) staining: Paraffin‐embedded tissue sections were incubated with primary antibodies against NKp44, CD8, and CD31, followed by horseradish peroxidase‐coupled secondary antibodies. Slides were washed with PBS and incubated with diaminobenzidine for 3–5 min to develop a dark brown color. To avoid bias during the image acquisition, we took images in a blinded manner. For quantification, 5 random 40 × magnification photographs were taken of each section. Cellular morphology was examined using an OLYMPUS IX73 Microscope (Tokyo, Japan), and assessments were conducted by a pathologist who remained blinded to treatment conditions. Images were obtained from five visual fields (upper, lower, left, right, and middle) on HE‐stained (40×) and IHC (40×) slides. The pathologist counted the positive expression of CD8, NKp44, and the number of micro vessels (CD31 positive) using Image Pro Plus 6.0 software (Media Cybernetics, USA).

### 2.5 Flow cytometry

Acquire single‐cell suspensions from immune organs and peripheral blood using established protocols. After rinsing the cells with PBS, reconstitute them in a staining buffer (PBS with 2% fetal bovine serum). The panel of antibodies included CD45‐APC‐Cy7, CD49b‐PE, CD3‐Percp‐Cy5.5, CD11c‐PE‐Cy7, MHC‐II‐APC. Subsequently, all antibodies were combined in a single staining tube and incubated for 40 min in the absence of light. Then, add 1 mL of erythrocyte lysate, gently blow and mix, react for 20 min at room temperature and avoid light, centrifuge at 1, 500 rpm for 5 min, and discard supernatant. After rinsing twice with 1 mL of PBS at 1, 000 rpm for 5 min, the supernatant was discarded, and the remaining sediment was resuspended in 500 μL of PBS for subsequent machine‐based detection (BD FACSAria II FlowCytometer 2 Laser Base Configuration).

### 2.6 Western blotting

The purified proteins from tumor tissue were extracted employing a tissue homogenizer and tissue protein lysate, subsequently subjected to separation on a 12% SDS‐PAG. Total protein was 40 μg/lane. Following electrophoresis, the proteins resolved within the gel were subsequently transferred onto PVDF membranes. The membranes underwent blocking with 5% nonfat milk in tris‐buffered saline with Tween‐20 (TBST) buffer at room temperature for 2 h, then incubated with primary antibody Bax (1:1000, 10995‐1‐AP, Proteintech), anti‐Bcl2 (1:1000, 10995‐1‐AP, Proteintech), anti‐Cleaved Caspase3 (1:1000, 9661T, Cell Signaling Technology), anti‐VEGF (1:1000, 19003‐1‐AP, Proteintech) and β‐actin (1:5, 000, abs132001, Absin) at 4°C overnight. The membrane was washed with TBST, goat anti‐rabbit IgG (1:50000, 111–035‐003, Jackson ImmunoResearch) and goat anti‐mouse IgG (1:20,000, 115–035‐003, Jackson ImmunoResearch) were used to incubate the membrane at room temperature for 1 h 30 min. The blots were washed with TBST and developed using an ECL substrate (Thermo Fisher Scientific, Waltham, MA, USA) and gel imaging system exposure (Bio‐Rad Laboratories, Hercules, CA, USA). The protein relative density was quantified using Image J software, with β‐actin or glyceraldehyde 3‐phosphate dehydrogenase (GAPDH) serving as the loading control.

### 2.7 Quantitative real‐time PCR (q‐PCR)

Total RNA was extracted from frozen tumor specimens utilizing Trizol (93289‐100ML, Sigma‐Aldrich; Merck KGaA) and cDNA was synthesized by reverse transcription (M1705, Promega, USA) in the reaction system of 25 μL. q‐PCR was performed in duplicate for each target gene using SYBR Green dye (Promega, USA) as the fluorophore. The analysis was carried out on an ABI7500 Detection System (Applied Biosystems). Gene expression levels were quantified employing the 2^‐ΔΔCt^ method, with GAPDH as the reference gene for normalization. The primer sequences for the target genes were custom synthesized by Genecreate Biotechnology Co., Ltd. A comprehensive list of the primer sequences can be found in Table 1.

**TABLE 1:** Primer sequences for quantitative q‐PCR.

Gene	Primer Sequence (5′‐3′)
IFN‐γ	F: CACCTGATTACTACCTTCTTCAG R: GTTGTTGACCTCAAACTTGG
IFN‐γ R	F: GTGCCTAAAGGGAAAGGTC R: TGAAATACGAGGACGGAGAG
TNF‐α	F: TTCTCATTCCTGCTTGTGG R: TTGGGAACTTCTCATCCCT
TNF‐α R	F: AGCATGTATACCCAGGTCTG R: GATCTCCACCTGGTCAGTG

*Note*: F: Forward; R: Reverse.

### 2.8 ELISA

Using the enzyme‐linked immunosorbent assay (ELISA) kits to detect the levels of mouse IL‐6 (MM‐0163M2, Meimian, China) and IL‐15 (ml160511, mlbio, China) concentrations in serum. The protocols were conducted in accordance with the guidelines provided by the manufacturer. The concentrations of target proteins were assessed using standard protein curves.

### 2.9 Statistical analysis

Each experiment was conducted independently, with a minimum of three repetitions to ensure robust data. The results are expressed as means ± standard deviation (SD). Statistical analyses were carried out using GraphPad Prism version 8 (GraphPad, La Jolla, CA, USA). Group comparisons were assessed using one‐way analysis of variance (ANOVA). Statistically significant outcomes were determined by a *p*‐value <0.05.

## 3 RESULTS

### 3.1 Combination of exercise training with doxorubicin effectively inhibited the progression of breast cancer in vivo

**FIGURE 2:**
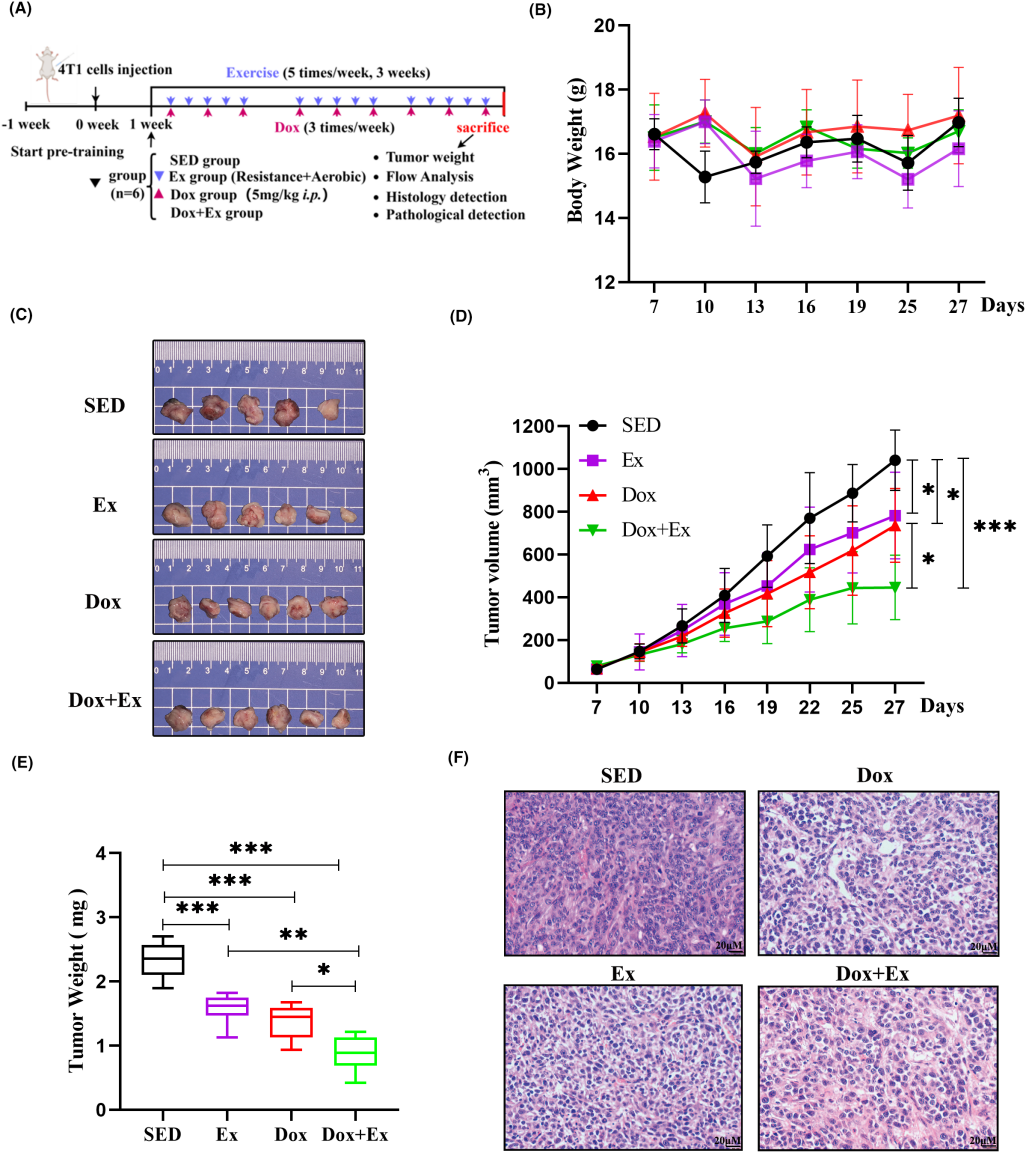
Combination of Exercise training with doxorubicin effectively inhibited the progression of breast cancer in mice model. (A) The flow chart of experiment. (B) Body weight of mice. (C) Typical images of tumor mass. (D) The tumor volume. (E) The tumor weight. (F) Typical images of H&E staining for tumor tissue (scale bars = 20 μm). For (B)–(E), *n* = 6 for each group (A mouse of SED group that died during the experiment was excluded). For (F), *n* = 3 for each group. Data are presented as mean ± SD. *p < 0.05, **p < 0.01, ***p < 0.001.

To explore if routine physical activity can collaborate with Dox in restraining the progression of breast cancer, we integrated resistance training with aerobic exercise to amplify the anticancer impact of physical activity. Our study utilized specific modeling techniques and intervention strategies (Figure 2A). As a result of this successful modeling, the mice maintained a stable appetite, and their weight remained within the normal range (Figure 2B). All breast cancer model mice had their tumors surgically removed, except for one tumor‐bearing mouse in the SED group who died midway (Figure 2C). Increasing exercise training significantly reduced tumor volume (Figure 2C,D) (*p* < 0.05) and tumor weight (Figure 2E) (*p* < 0.001) in the Ex group compared to the SED group. Additionally, the Dox group displayed notable reductions in tumor volume (*p* < 0.05) and mass (*p* < 0.001) as a positive control (Figure 2D–E). Notably, the decrease in tumor size and weight was more pronounced in the Dox + Ex group (Figure 2C–E) (*p* < 0.001), suggesting a potential improvement in therapeutic effect. Histological analysis revealed that tumor tissue in the SED group displayed no necrotic areas, deep nuclear staining, polygonal cell morphology, and vigorous cell proliferation. In contrast, both the Ex group and the Dox group exhibited a significant decrease in tumor cell density. In addition, obvious cell apoptosis and cell vacuolation, nuclear shrinkage, and cell number reduction were found in the tumor tissue from Dox + Ex groups (Figure 2F). These results suggest that the synergistic therapy of chemotherapy and exercise efficiently inhibits the growth of breast cancer.

### 3.2 Combination of Exercise training with doxorubicin effectively inhibited splenomegaly

**FIGURE 3:**
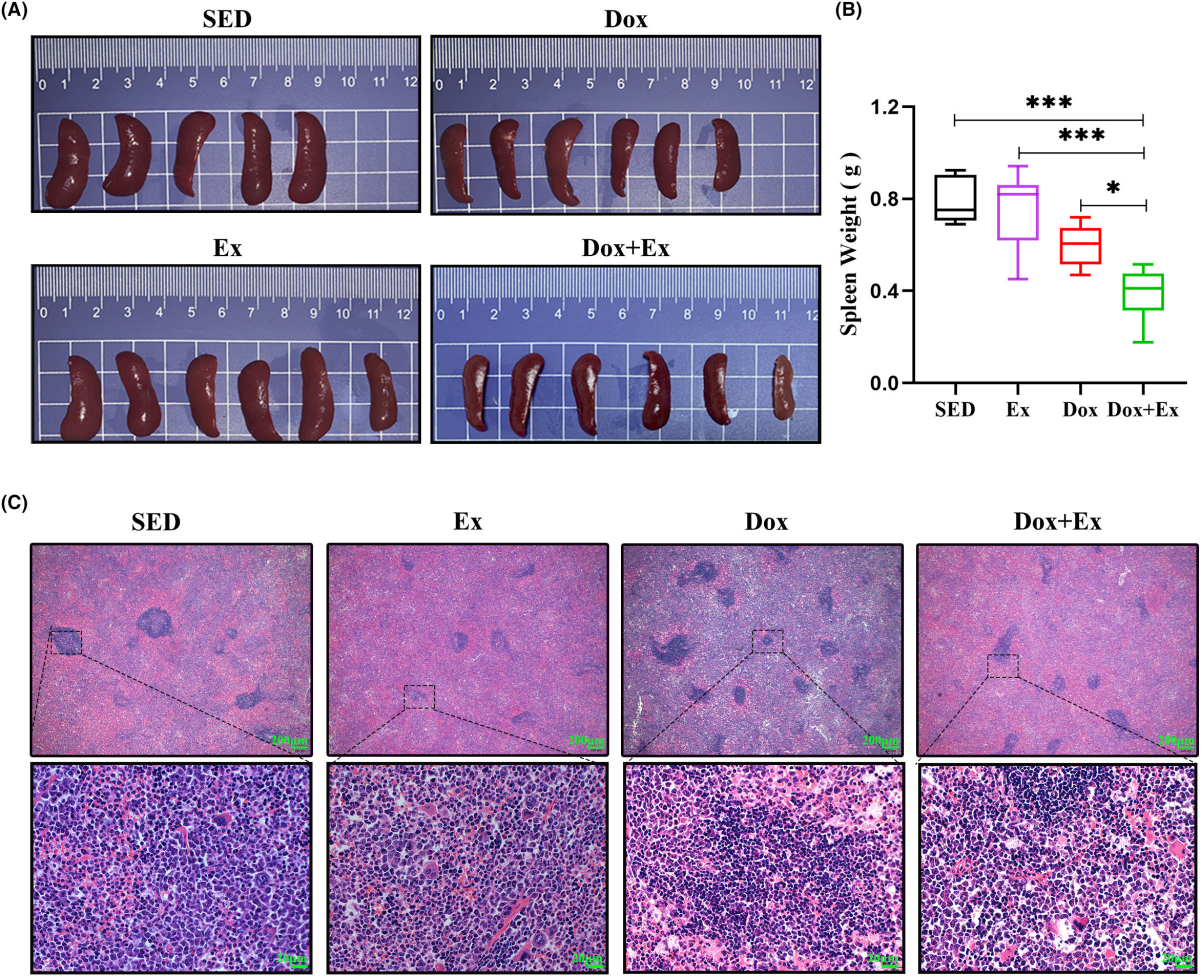
Combination of Exercise training with doxorubicin effectively inhibited splenomegaly. (A) Typical images of spleen. (B) Spleen weight, *n* = 6, (A mouse of SED group that died during the experiment was excluded), data are presented as mean ± SD, *p < 0.05, **p < 0.01, ***p < 0.001. (C) Typical images of H&E staining for mouse spleen tissue (scale bars = 20 μm).

Enlargement of immune cells associated with tumors has been documented to exhibit a positive correlation with the advancement of tumors.^17^ Splenomegaly, characterized by the enlargement of the spleen due to the proliferation of splenic granulocytes, including myeloid‐derived suppressor cells (MDSCs), is a notable phenomenon in mice bearing 4T1 tumors.^18^ The images display images of mouse spleens taken at the endpoint (Figure 3A). At the endpoint, the spleens of the SED group exhibited significant enlargement, while the reduction in spleen enlargement was not noticeable in the Ex group. The administration of Dox and Dox + Ex resulted in a decrease in the enlargement of the spleen induced by the tumor, respectively (Figure 3B). These findings demonstrate a marked reduction in splenomegaly induced by 4T1 tumors upon treatment with Dox. Histological assessment of the spleen was conducted using H&E staining. As illustrated in Figure 3C, pronounced splenocyte swelling and substantial accumulation of immune‐related cells were evident in the spleens of the SED group. The immune cell counts in the Ex group showed a slight decrease. In the Dox group and Dox + Ex group, there were no apparent signs of morphological alterations in splenocytes or the overall spleen structure.

### 3.3 Exercise training promoted the mobilization and redistribution of immune cells

**FIGURE 4:**
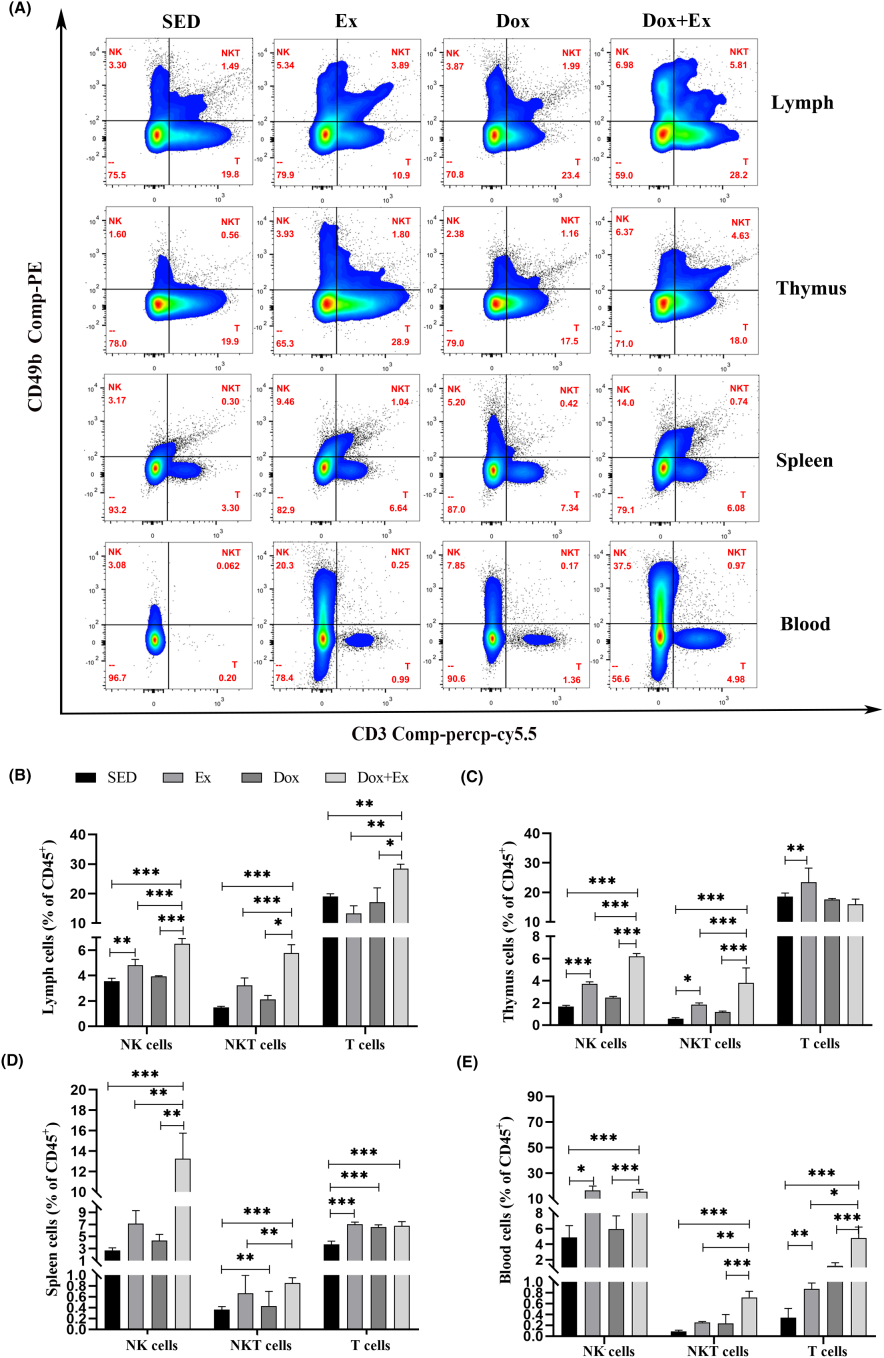
Exercise training promoted the mobilization and redistribution of immune cells. NK cells NKT cells and T cells in lymph, thymus, spleen and peripheral blood were detected by flow cytometry. (A) Strategy diagram of flow cytometry. Quantization of NK cells NKT cells and T cells in CD45^+^ cells of lymph (B), thymus (C), spleen (D), and peripheral blood (E). Data were presented as mean ± SD, *n* = 6, *p < 0.05, **p < 0.01, ***p < 0.001.

NK and T cells are vital components of the immune system, actively engaging in tumor surveillance and elimination throughout tumor development. Physical activity induces a swift mobilization of NK cells into the bloodstream, augmenting their responsiveness to cancer cells.^19^ To confirm the stimulation of immune cells through exercise training and doxorubicin, and to explore whether the two exert additive effects on immune activation, we performed flow cytometry analysis on lymphocytes from the peripheral blood, spleen, lymph, and thymus tissues of mice in each group (Figure 4A). The outcomes of flow cytometry revealed a notable rise in the proportion of NK cells in the Ex group compared to the SED group (*p* < 0.05). The NK cells elevation was not significant in the Dox group. The Dox + Ex group exhibited a substantial increase in the proportion of NK cells compared to the remaining three groups (*p* < 0.001) (Figure 4B–E). NKT cells represent a distinctive subset of T cells characterized by the presence of both T cell receptor (TCR) and NK cell receptors on their cellular membrane.^20^ Alterations in NKT cell proportions within the thymus, lymph nodes, and peripheral blood exhibited a similar trend to that observed in NK cells (Figure 4B–E). Exercise training also led to an increase in T cell proportions and stimulated their maturation across multiple immune organs. In the SED group, peripheral blood T cell counts were reduced, potentially as a consequence of heightened antigen burden and prolonged antigen exposure, contributing to pronounced T cell exhaustion.^21^ Our results indicate that exercise training can significantly improve T cell exhaustion (Figure 4A,E). Additionally, the proportion of NK cells and T cell did not show a significant change in the Dox group. These results suggested that the activation response to the immune system was mainly due to the benefits of exercise training.

### 3.4 The combination of exercise training with doxorubicin mobilized and redistributed cytotoxic immune cells through IL‐15 and IL‐6

**FIGURE 5:**
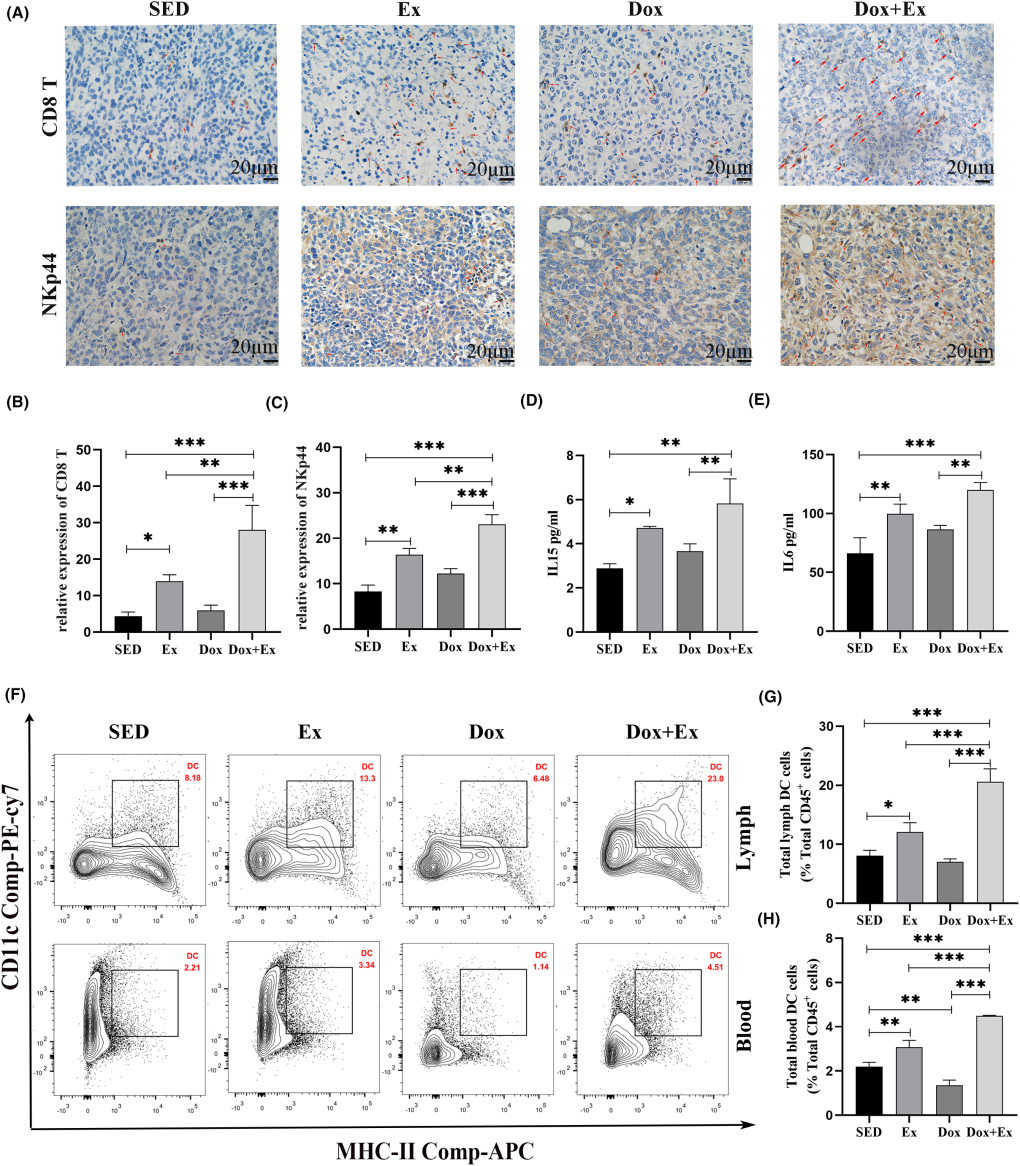
Combination of Exercise training with doxorubicin mobilized and redistributed NK cells and T cells through IL‐15 and IL‐6. (A) Typical images of immunohistochemical staining for CD8 and NKp44 in tumor tissue (scale bars = 20 μm). The relative intensity of CD8 expression (B) and NKp44 expression (C) was quantified. Concentrations of IL‐15 (D) and IL‐6 (E) in mouse serum. (F) DC cells of Lymph and thymus were analyzed by flow cytometry. The proportion of DC cells in CD45^+^ white blood cells of lymph (G) and peripheral blood (H) Data were presented as mean ± SD, *n* = 6, *p < 0.05, **p < 0.01, ***p < 0.001.

Exercise‐induced physiological stress triggers the release of IL‐6 and IL‐15, two cytokines known to activate and expand NK cells.^22^ IL‐15 acts as a key regulator by enhancing NK cell proliferation and cytotoxic activity, while IL‐6 is perceived to boost NK cell activation and migration. Consequently, an ELISA assay was employed to assess the concentrations of IL‐15 and IL‐6 in the serum of mice. As Figure 5D,E demonstrate, the levels of IL‐15 and IL‐6 in the serum of the Ex group mice and Dox + Ex group mice were notably higher compared with the SED group mice (*p* < 0.01). Furthermore, in the serum of the Dox + Ex group, the concentrations of IL‐15 and IL‐6 increased by 49% and 55%, respectively, in comparison to the SED group. Subsequently, IHC staining was employed to quantify the degree of infiltration of CD8^+^ T cells and NK cells in tumor tissues. The results indicated a significant rise in the expression levels of CD8 and NKp44 in the Ex group. A significantly higher proportion of CD8^+^ T cells and NK cells were observed in the Dox + Ex group compared to both the Ex and Dox groups (*p* < 0.01) (Figure 5A–C). This suggests that exercise elevates the quantity and efficacy of cytotoxic immune cells, likely related to the amplified levels of IL‐15 and IL‐6. In the tumor microenvironment, the function and activity of DCs may be inhibited, fostering immune escape. However, NKp44 was discovered to invigorate DC activation and functional enhancement. Particularly, cytokines released by NK cells can spur the maturation and antigen‐presenting capacity of DCs while amplifying their ability to produce immunostimulatory molecules.^23^ As shown in Figure 5F–H, the relative proportion of DCs in the mouse lymph and peripheral blood of other groups was significantly elevated compared with the SED group and Dox group (*p* < 0.01), and the proportion of DCs was highest in the Dox + Ex group (*p* < 0.001). These findings indicate that physical activity triggers the reorganization of immune cells, enhancing the anti‐tumor impact of Dox.

### 3.5 Exercise training mitigated the cardiotoxicity induced by doxorubicin and suppressed tumor angiogenesis

**FIGURE 6:**
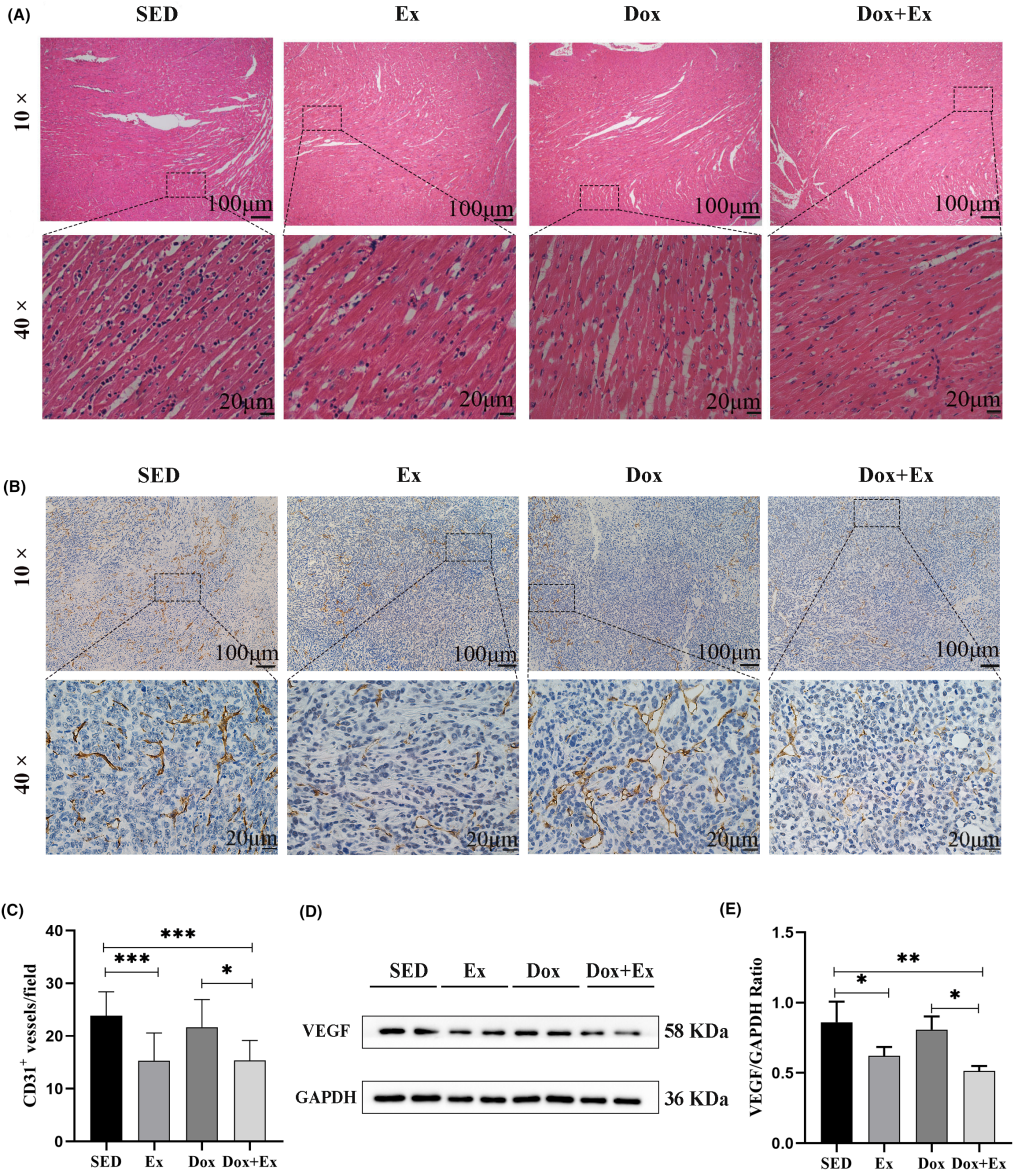
Exercise training improved the cardiotoxicity caused by doxorubicin and inhibited tumor angiogenesis. (A) Typical images of H&E staining for mouse heart tissue (scale bars = 20 μm). (B) Representative images of CD31 immunohistochemical staining in tumor sections (scale bars = 20 μm). (C) Quantification of the number of CD31^+^ vessels in tumor sections, data were presented as mean ± SD, *n* = 5. (D) Representative bands of VEGF expression were detected by Western blot. (E) Quantitative statistics of relative expression of VEGF, data were presented as mean ± SD, *n* = 3. *p < 0.05, **p < 0.01, ***p < 0.001.

Most chemotherapeutic drugs, utilized in clinical practice, have non‐specific target cells, leading to detrimental side‐effects due to the indiscriminate attack on normal cells while terminating tumor cells. For example, doxorubicin, a cell‐cycle non‐specific anticancer drug primarily induces cardiotoxicity.^19^ To evaluate the capability of aerobic exercise intervention in alleviating doxorubicin‐induced cardiotoxicity, immunohistochemical analyses were performed on cardiac tissues. Findings revealed that myocardial cell morphology in both SED and Ex groups appeared normal, with neatly arranged myocardial fibers uniformly stained. Contrarily, in the Dox group, myocardial fibers were disarrayed, their diameter increased and cytoplasm staining uneven; moreover, myocardial fibers exhibited swelling, vacuolation, degeneration, and even necrosis. However, the deleterious heart tissue alterations in the Dox + Ex group were markedly attenuated compared to solely Dox‐treated group, as depicted in Figure 6A. These findings imply that exercise can alleviate the cardiotoxic side‐effects of doxorubicin treatment. To further discern the regulation of tumor angiogenesis by aerobic exercise intensity, we evaluated CD31 expression in tumor tissue blood vessels. Utilizing both IHE and Western blotting techniques to stain and quantify CD31 and VEGF expressions, we assessed the degree of tumor angiogenesis and the potential inhibitory effect of exercise on tumor proliferation mediated through angiogenesis. Compared to the SED group, our results indicate a significant decrease in angiogenesis within the tumor tissue after exercise (*p* < 0.001). Additionally, angiogenesis was distinctly diminished in the Dox + Ex group relative to the Dox group (*p* < 0.001) (Figures 6B–E).

### 3.6 Combination of exercise training with doxorubicin more effectively activated the mitochondrial apoptosis of breast cancer cells

**FIGURE 7:**
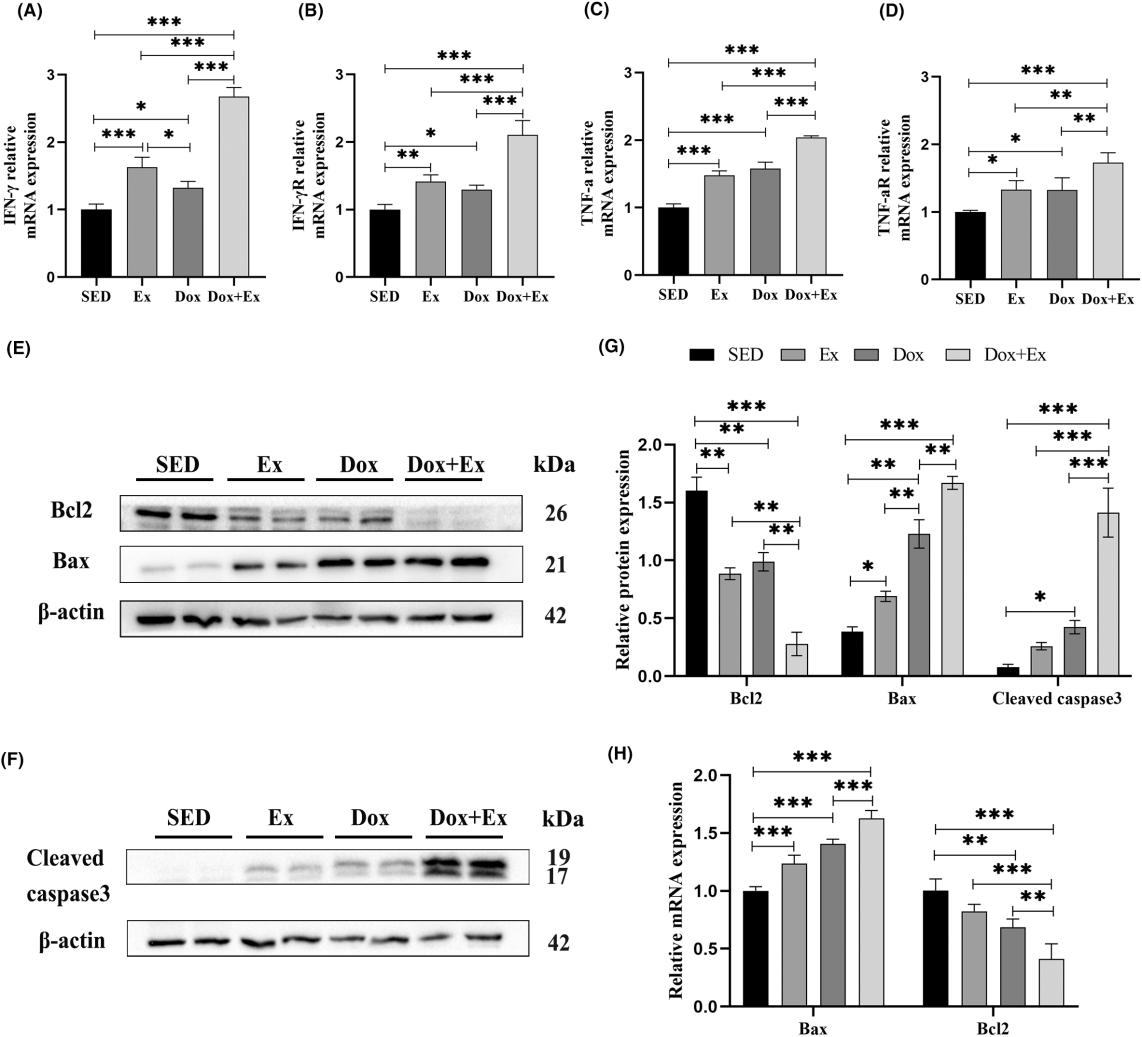
Combination of Exercise training with doxorubicin more effectively activated the mitochondrial apoptosis of breast cancer cells. IFN‐γ (A), IFN‐γ R (B), TNF‐α (C), TNF‐α R (D) mRNA expression was analyzed by q‐PCR, data were presented as mean ± SD, *n* = 3. Representative bands of Bax, Bcl2 (E), and Cleaved caspase3 (F) expression were detected by Western blot. (G) Quantitative statistics of relative expression of Bax, Bcl2, and cleaved caspase3, data were presented as mean ± SD, *n* = 3. (H) Bax and Bcl2 mRNA expression was analyzed by q‐PCR, data were presented as mean ± SD, *n* = 3. *p < 0.05, **p < 0.01, ***p < 0.001.

As indicated by previous observations, regular physical exercise impedes tumor progression by stimulating the activation of cytotoxic immune cells and remodeling the tumor microenvironment. This is especially apparent in the increased infiltration rates of NK cells and CD8^+^ T cells within tumor tissues. Cytotoxic immune cells induce cancer cell apoptosis through various molecular pathways. These pathways include releasing perforin, granzyme, interferon‐gamma (IFN‐γ), tumor necrosis factor‐alpha (TNF‐α), and other factors that initiate the tumor cell apoptosis pathway.^24^ The q‐PCR data demonstrated a substantial upregulation in the expression levels of IFN‐γ and TNF‐α, along with their respective receptors, within both the Ex and Dox groups in comparison to the SED group (*p* < 0.001). Notably, the expression levels of TNF‐α and IFN‐γ and their receptors were significantly higher in the Dox + Ex group as compared to all other groups (Figure 7A–D). Furthermore, Western blotting analysis unveiled notable alterations in the expression of apoptosis‐related proteins. In comparison to the SED group, the Ex, Dox, and Dox + Ex groups exhibited a significant increase in the levels of Bax and Cleaved caspase‐3 proteins, concomitant with a significant decrease in Bcl2 expression. Importantly, the changes observed in apoptosis‐related protein expression were more pronounced in the Dox + Ex group relative to the other groups (Figure 7E–G). The trend in the mRNA levels of Bax and Bcl2 paralleled these findings (Figure 7H). These results suggest that the enhanced delay in breast tumor growth due to the synergy between exercise and doxorubicin might be mediated by the mitochondrial apoptotic pathway.

### 3.7 Discussion

Several clinical studies substantiate the idea that physical activity diminishes cancer occurrence, hinders recurrence, and amplifies the therapeutic outcomes of anticancer treatments.^25^ In addition to the health benefits, the impact of physical exercise in augmenting NK cell functionality during breast cancer therapy has been demonstrated through preclinical evidence.^26^ Nonetheless, some clinical investigations have indicated that exercise does not exert a substantial influence on the survival rates of cancer patients receiving chemotherapy.^27^, ^28^ The varying impact of physical exercise on the therapeutic effects of chemotherapy may be related to cancer type, drug type, exercise style, and intensity.^29^, ^30^ Both aerobic exercise and resistance exercise are beneficial to the body; aerobic exercise primarily improves cardiovascular and immune system functions, while resistance exercise primarily enhances muscle strength and exercise capacity.^31^ In this study, high‐intensity aerobic exercise and resistance training were performed using a rotating rod treadmill and forced stair climbing in a breast cancer mouse model. Consistent with previous studies,^32^ inhibiting the increase in tumor volume and weight suggests that exercise can significantly suppress the rapid growth of cells in vivo. It is noteworthy that the combined treatment of doxorubicin and exercise has a better effect on tumor inhibition than doxorubicin therapy alone. This result further supports the idea that physical exercise can significantly improve the efficacy of chemotherapy in a breast cancer model.^33^

Furthermore, the outcomes of flow cytometry and IHE analyses illustrated that physical exercise promoted the mobilization of lymphocytes in immune organs, concurrently enhancing the proportion and tissue infiltration of cytotoxic immune cells. Following the exercise regimen, the body synthesizes signaling molecules referred to as “motility factors,” which exert a pivotal influence on immune system functionality. This process involves adrenaline secretion and stimulation of NK cells and CD8^+^ T cells within blood vessels.^34^ Physical activity also triggers the secretion of substances, such as myokines IL‐15 or IL‐6 within muscle tissue, initiating the redistribution of immune cells and eventual infiltration of the tumor site to eradicate tumor cells.^35^ Our experimental results confirmed that levels of IL‐15 or IL‐6 in the peripheral blood of breast cancer model mice subjected to exercise intervention were significantly elevated. Concurrently, IFN‐γ and TNF‐α levels, as well as NK cell and CD8^+^ T cell infiltration in tumor tissues were increased. Notably, such an effect was absent in the group treated with doxorubicin alone. These discoveries provide additional corroboration for the theory that physical activity engenders an anti‐tumor influence through the facilitation of immune cell mobilization and repositioning. Additionally, our findings indicate that exercise regimens may augment the activation and functionality of DCs by fostering the synthesis of immunostimulatory molecules, thereby potentially enhancing the anti‐tumor response when combined with doxorubicin therapy. The mitochondrial apoptosis (mt‐apoptosis) pathway assumes a pivotal role in enabling cytotoxic immune cells to efficiently eliminate neoplastic cells.

NK cells induce mt‐apoptosis in cancer cells, and the mitochondrial state affects cancer cell vulnerability to NK‐mediated destruction.^36^ Our study revealed that both doxorubicin and exercise activate the mt‐apoptosis pathway in tumor cells, with the combined impact of both interventions proving more significant. This finding suggests that exercise activates the mt‐apoptosis pathway in tumor cells through cytotoxic immune cell stimulation, thus enhancing doxorubicin's tumor‐inhibiting effect.

Our research highlights the diverse advantages of physical activity, not only in enhancing general well‐being but also in alleviating the adverse effects of cancer treatments. Indeed, the cardiotoxicity caused by doxorubicin accumulation within off‐target tissues is a major concern in cancer therapy,^37^ and it is remarkable that exercise training could aid in improving the underlying morphological abnormalities of myocardial fibers induced by such treatment. It is also interesting to note the influence of exercise on intratumoral angiogenesis, a crucial aspect of tumor growth and expansion.^38^ There are conflicting effects of exercise on tumor angiogenesis, which may be related to the paradoxical effect of intratumoral angiogenesis.^39^, ^40^, ^41^, ^42^, ^43^ The previous study importantly points out that exercise may reduce the endothelial and naïve T cell adhesion mediated by CD31 and its ligand, and may inhibit the immunosuppressive CD4 infiltration in tumor tissues, thereby limiting the growth and spread of the tumor.^44^ However, CD31 may be involved in CD45RA CD4 T cell function to induce inhibitory T cell production.^45^ Although we observed a vascular‐like expression of CD31, the changes of CD31 in tumor tissues may be caused by modification of other cell populations. Therefore, necessitates deeper understanding is still needed to observe the complex interplay of vascular‐like CD31 expressions as linked to the changes in tumor tissues in future. Also noteworthy is that physical activity has the potential to modulate the concentration of angiogenic factors within the tumor microenvironment.^46^ The decrease in VEGF expression level with exercise furtherly suggests possible inhibitory effects on angiogenesis in tumor tissues.

Overall, the intersection between exercise and chemotherapy is a field with vast unexplored potential, and this research opens an essential gateway toward the understanding of such a relationship. Noteworthy limitations of our study include our reliance on measurements at a single time point, which hampers our dynamic observation of exercise‐induced tumor mechanisms. Rigorous clinical trials in the future will be indispensable to validate the safety and efficacy of exercise as a complementary approach to cancer treatment, in conjunction with the development of individualized exercise regimens. The potential realization of this paradigm shift has the potential to transform our understanding and implementation of cancer treatment.

## 4 CONCLUSION

This study represents the inaugural exploration into the impact of aerobic exercise in conjunction with resistance training on the efficacy of chemotherapy within a murine model of breast cancer. The findings are intriguing, especially on how an exercise regimen could enhance the efficacy of chemotherapy via boosting the activity of cytotoxic immune cells and promoting their infiltration into tumor tissues, a significantly promising revelation in the quest for effective cancer treatment. Furthermore, the initiation of the mitochondrial apoptosis pathway within tumor cells by these activated immune cells is another key aspect that underscores the importance of exercise. The results of this study also support that the combination of exercise and chemotherapy reduces doxorubicin‐related cardiotoxicity and inhibits tumor angiogenesis, thus further amplifying the tumor suppressive effect of doxorubicin. Consequently, the combination of exercise training with chemotherapy presents a highly promising avenue for the prevention and treatment of breast cancer.

## AUTHOR CONTRIBUTIONS

**Bingqing Qin:** Data curation (equal); formal analysis (equal); funding acquisition (equal); investigation (equal); methodology (equal); validation (equal); visualization (equal); writing – original draft (equal); writing – review and editing (equal). **Zhongshi He:** Funding acquisition (equal); project administration (equal); supervision (equal); visualization (equal). **Lixia Xie:** Data curation (equal); formal analysis (equal); resources (equal); software (equal). **Shenglan Feng:** Data curation (equal); formal analysis (equal); software (equal); visualization (equal). **Junjie Ye:** Conceptualization (equal); data curation (equal); resources (equal); validation (equal). **Jianjun Gui:** Data curation (equal); investigation (equal); software (equal); visualization (equal). **Xiaodong Sun:** Data curation (equal); funding acquisition (equal); investigation (equal); writing – original draft (equal); writing – review and editing (equal). **Ming Sang:** Data curation (equal); funding acquisition (equal); methodology (equal); supervision (equal); writing – review and editing (equal).

## FUNDING INFORMATION

This investigation was supported by the Experimental Animal Resources Development and Utilization Project of Hubei Province of China (2020DFE025), the Innovative Team Project from the Institute of Medicine and Nursing at Hubei University of Medicine (2017YHKT02), the Foundation of Health Commission of Hubei Province (WJ2023M161), the Scientific and Technological Project of Xiangyang City of Hubei Province (2022YL27B), and Innovative Research Program for Graduates of Hubei University of Medicine (YC2022003, YC2022008, YC2023006). Open Research Fund Program of the State Key Laboratory of Virology of China (2023KF001).

## CONFLICT OF INTEREST STATEMENT

The authors declare no conflicts of interest.

## ETHICS STATEMENT

This study was approved by the Ethical Committee for Animal Experimentation of Xiangyang No. 1 People's Hospital (approval number XYYYE20220030).

## Data Availability

The data supporting this study's findings are available from the corresponding author upon reasonable request.
